# Mutations in the Global Transcription Factor CRP/CAP: Insights from Experimental Evolution and Deep Sequencing

**DOI:** 10.1016/j.csbj.2019.05.009

**Published:** 2019-06-18

**Authors:** Pernille Ott Frendorf, Ida Lauritsen, Agnieszka Sekowska, Antoine Danchin, Morten H.H. Nørholm

**Affiliations:** a Novo Nordisk Foundation Center for Biosustainability, Technical University of Denmark, Kemitorvet B220, DK-2800 Kgs. Lyngby, Denmark; b Institut de Cardiométabolisme et Nutrition, CHU Pitié-Salpêtrière, 47 boulevard de l'Hôpital, 75013 Paris, France; c Institut Cochin, INSERM U1016, CNRS UMR8104, Université Paris Descartes, 24 rue du Faubourg Saint-Jacques, 75014 Paris, France

**Keywords:** cAMP receptor protein, Carbon catabolite repression, Global transcriptional regulation, Experimental evolution

## Abstract

The *Escherichia coli* cyclic AMP receptor protein (CRP or catabolite activator protein, CAP) provides a textbook example of bacterial transcriptional regulation and is one of the best studied transcription factors in biology. For almost five decades a large number of mutants, evolved *in vivo* or engineered *in vitro,* have shed light on the molecular structure and mechanism of CRP. Here, we review previous work, providing an overview of studies describing the isolation of CRP mutants. Furthermore, we present new data on deep sequencing of different bacterial populations that have evolved under selective pressure that strongly favors mutations in the *crp* locus. Our new approach identifies more than 100 new CRP mutations and paves the way for a deeper understanding of this fascinating bacterial master regulator.

## 1 Introduction

**Fig. 1:**
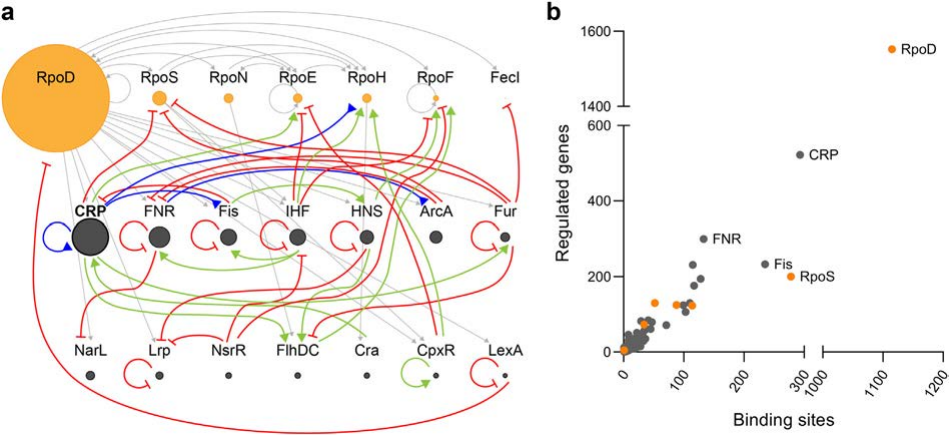
Network of global transcription factors and sigma factors in *Escherichia coli*. Illustrated is a) The sigma factors (orange circles) and the 14 biggest regulators by regulon size (grey circles). The size of the circles is directly proportional to the sigmulon or regulon size by number of genes directly affected (RegulonDB 04-03-2019) [19]. Arrows designate regulation of regulator expression; sigma factor transcription (grey arrows), activation (green, arrowheads), repression (red, perpendicular line ends) or dual regulation (blue, reverse arrowheads) [26]. b) The sigma factors (orange circles) and 207 regulators (grey circles) of *E. coli* plotted by the number of promoters or binding sites recognized, respectively, and the number of genes directly affected (RegulonDB 04-03-2019) [19].

Studies of CRP date back to the earliest days of molecular biology, shortly after the model for negative *lac* gene regulation was presented [25]. In the decade following Jacob and Monod's groundbreaking discoveries, several reports on positive gene regulation were published including catabolite activation by CRP [14,15,69]. CRP is mostly known for its global regulatory role in carbon catabolism in the model bacterium *Escherichia coli (E. coli)*: In the absence of readily metabolized carbon sources such as glucose, the enzyme adenylate cyclase is activated, producing cyclic AMP (cAMP) from ATP. cAMP binds and activates CRP, increasing the affinity for DNA, which in many cases activate operons involved in the utilization of alternative carbon sources such as lactose and maltose. However, CRP can also repress gene expression and has been shown to regulate hundreds of genes in the *E. coli* genome, earning it the status of “global” or “master” regulator (Fig. 1) [31]. In fact, beyond the many specific binding sites experimentally validated in *E. coli*, CRP exhibits unspecific DNA binding affinity and together these observations points towards a role more akin to that of a nucleoid-associated protein involved in the organisation of the bacterial chromosome [55].

The first experimentally solved three-dimensional structure of CRP bound to cAMP was published in 1981 [32], but for many years the absence of an experimentally determined structure of apo-CRP hindered the understanding of the conformational changes that occur upon cAMP binding [18,27]. Presently, only one crystal structure [62] and one NMR structure [39] of wildtype apoCRP have been published. Both structures indicate that large structural rearrangements take place for DNA binding to occur. These include reorientation of the DNA-binding domain and stabilization of the backbone helix, but the two different structures do not agree on the orientation of the C-terminal domain. This observation, and the limited number of published apo CRP structures, suggests that apoCRP may be unstable due to flexibility of the C-terminal domain [44].

**Fig. 2:**
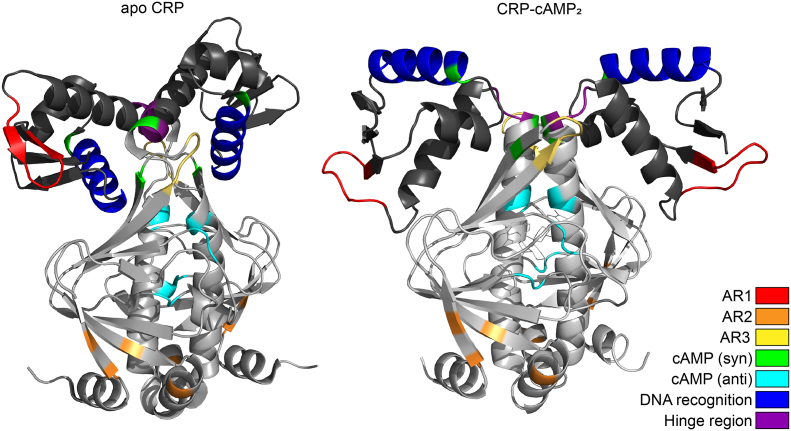
Illustration of functional domains in a closed and an open conformation of CRP. The locations of functionally important CRP domains in the tertiary structure of apo-CRP (left) and CRP-cAMP_2_ (right). The protein structures are from Protein Data Bank entries 3FWE (apo-CRP) and 1ZRC (CRP-cAMP_2_) and were modified using the PyMOL Molecular Graphics System, Version 2.3 Schrödinger, LLC.

CRP is a 45 kDa homodimer (Fig. 2), with each monomer consisting of 209 amino acids in two separate domains. The larger N-terminal domain (residues 1–138) binds the allosteric effector cAMP in the *anti-*conformation (residues 71, 72, 82, 83, 127 and 128) with reported binding constants in the range of 1–28 μM [2,13,14,22,30,56]. The C-terminal domain (residues 139–209) houses the DNA-recognition helix (residues 181–193) as part of a helix-turn-helix (HTH) motif [6]. The N- and C-terminal domains are connected by a hinge region (residues 135–138). In the absence of ligand, CRP exists in a closed conformation (Fig. 2, left), where the HTH motif is secluded inside the C-terminal domain. When cAMP binds the main binding site, allosteric change stabilizes an open complex (Fig. 2, right), resulting in the HTH motif protruding from the surface of the protein, thereby enabling DNA binding. A secondary effector binding site binds cAMP in its *syn*-conformation (residues 58, 135, 180) with binding constants in the millimolar range likely to be of limited physiological relevance [30,38]. In the presence of excess cAMP, both cAMP binding sites in one monomer are occupied, resulting in a lower DNA-binding affinity [38].

When CRP binds, the DNA is bent 90°, which likely significantly affects protein-protein and protein-DNA interactions in promoter regions [17,28,47,61] and several studies suggest that CRP is involved both in recruiting the RNA polymerase and in post-recruitment regulation [7,29,35,41]. In the case of transcriptional activation, regulation is mediated by direct interactions with the RNA polymerase holoenzyme through so-called activating regions (ARs). AR1 (residues 156–164) interacts with the αCTD domain [36,46,[63], [64], [65], [66]], AR2 (residues 19, 21, 96 and 101) with the αNTD domain [35,58] and AR3 (residues 52–55 and 58) with the sigma factor [4,41,42,[58], [59], [60]].

Complementary to structural and biochemical studies, evolved and rationally engineered mutations are imperative when elucidating protein structure and function. In this respect, CRP is probably one of the best studied transcription factors. For several decades, mutant generation has been applied to study the physiological role and molecular mechanism of CRP [37,45]. A comprehensive list of mutations identified in CRP was previously assembled [37], but to our knowledge the different approaches towards obtaining these CRP mutants have not been reviewed previously. In light of recent high throughput sequencing (HTS) approaches applied to *crp* mutants [48], we here aim at presenting a short overview on the more than 40 years of exploration of the CRP mutational space and in addition provide complementary new data on deep sequencing of experimentally evolved *crp*, obtained in aging bacterial colonies. An overview of all CRP mutants reviewed here is shown in Tables S1 and S2.

## 2 Evolved and Engineered Mutations in *crp*

The outcome of an evolution experiment is dependent on the mutational space available under the given experimental conditions, the genotype of the organism and the selection pressure applied. Evolution of CRP has been pursued by inducing mutagenesis using *e.g.* UV radiation or chemical reagents [33,45] or by directly targeting the *crp* gene with error-prone PCR [67], but several studies have also relied on spontaneously arising mutants [9,43,48,50]. In most studies, the CRP variants were generated in a cAMP-deficient production strain (Δ*cya*) and screened for mutations enabling fermentation of a carbon source such as lactose or maltose. Such cAMP suppressor mutations were termed *csm* [33] and CRP variants called CRP* [45] or CRP^i^ [5].

In the 1970's, early after the discovery of CRP, researchers began isolating *csm,* CRP* [5,12,45,51,54] and defective *crp-* mutants [3] mostly under conditions of induced mutagenesis. However, the exact molecular nature of these mutants was unknown for some time due to the lack of DNA sequencing and amplification technologies.

In 1985, Aiba and colleagues published a paper where they had exposed a plasmid-borne *crp* gene to UV-radiation and selected for lactose utilization in a Δ*cya* strain. The obtained *crp* mutations in the isolated strains caused amino acid substitutions in positions 53, 62, 141, 142 and 148 of the CRP protein [1]. It was noted that positions 53 and 62 were in vicinity of the cAMP binding site, but that the phenotypes differed in that only CRP D53H was activated by the alternative cyclic nucleotide cGMP. The role of position 53 being located in the AR3 region, possible interacting with sigma factors, was not discussed in this work as AR3 was unknown at this time. Amino acids 141, 142 and 148 are part of the D-α-helix and it was speculated that they were critical in the allosteric transition, from the N-terminal domain to the DNA binding C-terminal domain, normally caused by binding of cAMP.

Around the same time, Garges and Adhya used *crp*-carrying phages for infection and growth in a mutator *E. coli* strain for CRP mutant generation [21]. Phages carrying mutagenized *crp* variants were isolated as positive lactose utilizing plaques in an Δ*cya* background. The detected mutations were in positions 72, engaged in cAMP binding, and again in D-α-helix residues 141, 142 and 144.

The following year, Harman and colleagues sequenced and characterized three CRP* mutants that previously were selected by different methods [23,33,40,45]. The CRP mutation A144T in the D-α-helix was again identified – in this case from a Δ*cya* strain selected on xylose as carbon source. A T127I mutation in the cAMP binding site was identified in combination with Q170K from a strain that complemented a CRP binding site mutation designated L8 in the *lac* promoter [45]. This double mutant showed a CRP* phenotype and was activated by cGMP, although the physiological relevance of the latter was questioned by Harman and colleagues. The individual effects of the 127 and 170 mutations were not explored further in this work. Finally, the mutation L195R was evolved in the Δ*cya*, *crp T127I, Q170K* mutant background and the extra mutation enabled growth on arabinose in the absence of cAMP [40]. The authors suggested that the increased positive charge of this L195R mutation in the DNA binding domain caused an increase in the affinity for DNA.

In a follow-up study by Garges and Adhya, CRP* suppressor mutants, causing a loss of the G141S and A144T CRP* phenotypes on lactose, were identified as T127A and R169C/E171G, respectively [20]. In case of the G141S CRP* mutant, it is perhaps not surprising that a mutation near the cAMP binding (T127A) can neutralize cAMP independence. Similarly, it was noted that amino acids near the two mutations in positions 169 and 171, identified in the CRP A144T mutant background, were previously suggested to interact with the amino acid Y63 near the cAMP binding site, but also could be in direct interaction with the DNA [57].

Mutations in positions 141, 141 and 144 again occurred in a study that described the selection of CRP* mutants based on growth on lactose in a Δ*cya* mutant background [53]. In this case, two different mutations in position 144, A144T and A144E, were found in combination with T28K. These two combinations were found to be toxic when expressed on a multicopy plasmid, whereas two other D-helix mutations T140K and G141D were tolerated in high copy.

Another broad category of CRP mutations, more generally termed *positive control mutants (pcm)*, were selected for their inability to induce transcription while retaining binding to specific CRP DNA binding sites. The first attempt at creating CRP *pc* mutants introduced the mutations E171Q, E171K and Q170K based on similarity to the lambda repressor, but these caused different effects at different promoters [4,24]. A more clear *pcm* phenotype was observed with the mutation CRP H159L in AR1 and second site revertants was identified as K52N and K52Q in AR3 [4]. Eschenlauer and Reznikoff screened for CRP mutants that repressed the *gal* promoter but had lost their ability to induce the lac promoter [16]. This way, they identified mutations in cAMP binding position 72, and in position 162 in the AR1 region. In a similar study by Zhou and co-workers, plasmid-harbored *crp* genes were mutagenized by error-prone PCR and screened in an engineered Δ*cya* strain for defective ribose fermentation, while retaining the ability to repress a modified *lac* promoter [66]. The identified mutations were in positions 156, 158, 159, and 162 that are all part of AR1. Finally, Niu and co-workers identified mutations in the AR1 region that could not activate transcription of Class I and II CRP-dependent promoters as well as the mutations H19L, H19Y, H21L and K101E in the AR2 region that were only defective in Class II promoter activation [35].

Experimental evolution of CRP has also addressed the complex interplay between CRP and the CytR transcription factor. A CytR-repressed *tsx* promoter construct was screened in combination with a mutagenized *crp* plasmid library for CRP variants that were dominantly activating the promoter. This approach identified mutations in positions 17, 18, 108 and 110 in CRP – all in the vicinity of AR2 [52].

Recent work has again explored CRP mutants that evolve spontaneously under different selection regimes. Sievert and co-workers observed that by growing an *E. coli* strain in high levels of the (CRP-dependent) carbon source xylose, the CRP G141D mutation again was found to evolve, promoting increased xylose utilization and growth rate [50]. In adaptive laboratory evolution for improved fitness in minimal medium supplied with lactate, the CRP mutations L150Q and I165T evolved [9]. These are both located near AR1 and presumably cause a changed interaction with the RNA polymerase.

**Fig. 3:**
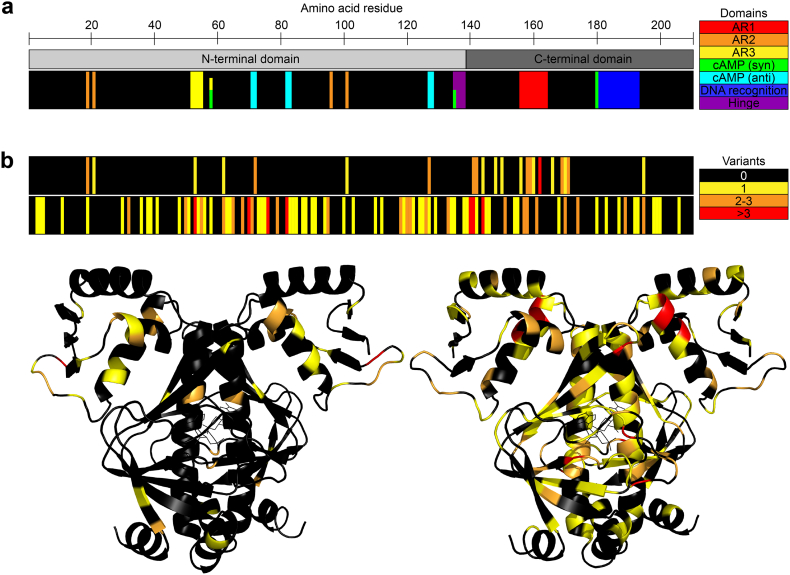
Illustration of adaptive mutations identified in CRP a) The locations of functionally important CRP domains in the primary amino acid sequence. b) Locations of mutations identified in the primary and tertiary structure of CRP in previous studies (upper black bar and structure to the left) and in our laboratories by deep sequencing (lower black bar and structure to the right). The number of variants identified per residue is colour coded (black: 0 variants, yellow: 1 variant, orange: 2–3 variants, and red: more than three variants).

In summary, adaptive mutations identified in the previous four decades of CRP studies (Fig. 3) occur predominantly in the cAMP binding site, the D-α-helix, and in the RNA polymerase activating domains AR1 and AR2. These three categories are intuitively easy to understand as they likely either directly affect ligand binding, ligand-induced allosteric transitions, or the productive interaction with the core RNA polymerase, respectively. Mutations around position 170 are also frequently observed but are more difficult to interpret and have been discussed both to directly affect interactions with cAMP and the DNA.

To supplement *crp* mutants that evolve under selective pressure, the advent of PCR enabled hypothesis-driven site-directed mutagenesis exploring the role of specific amino acid residues in the different domains of the CRP protein. As summarized in Table S2, these are often studied in areas where mutations evolve naturally (the cAMP-*anti* binding pocket, the D-α-helix and the activating regions AR1 and AR2), but have also explored mutations in *e.g.* the hinge region that connects the C- and D-helices, AR3, and the DNA binding domain in the C-terminus.

In an experiment designed to study adaptation when bacteria age and starve over two months, our recent work identified a large number of CRP mutants. By plating a Δ*cya E. coli* strain on MacConkey agar supplied with the CRP-dependent carbon source maltose, mutant red “*papillae*” - or “secondary colonies” - appeared that were able to use the extra carbon source. 96 mutants were selected for genome sequencing based on their different CRP* phenotypes and their temporal appearance. In addition, an additional approximately 500 mutants had their *crp* loci sequenced by PCR amplification and Sanger sequencing [48]. 35 different missense mutations were identified in *crp.* Seven of the identified mutations, S62F, T127I, G141D, G141S, A144T, A144E and L195R were previously observed in adaptive evolution studies and have all been classified as CRP*. Other *crp* mutations have not been identified elsewhere, including P110Q, L134M, T140P/R/K, A144K, G162S and M189K. The CRP mutant S62Y has not been identified before. However, the substitution of serine to phenylalanine (S62F) has been observed by induced mutagenesis and screening on lactose previously [1]. The Q170K mutation was always observed in combination with an additional *crp* mutation such as T140R, A144T/E or M198K [48]. Interestingly, a similar trend was observed in the study by Harman and co-workers in which the Q170K mutation was paired with T127I or T127I and L195R [23].

## 3 Deep Sequencing of CRP in Aging Bacterial Populations

We reasoned that we could obtain deeper insights into the mutational space of CRP by growing a large number of different bacterial colonies, followed by deep sequencing of the *crp* locus. To this end, we followed the same workflow as previously described [48], but now isolated DNA from more than 500 colonies per plate at different time points, followed by PCR amplification of *crp* and HTS.

**Fig. 4:**
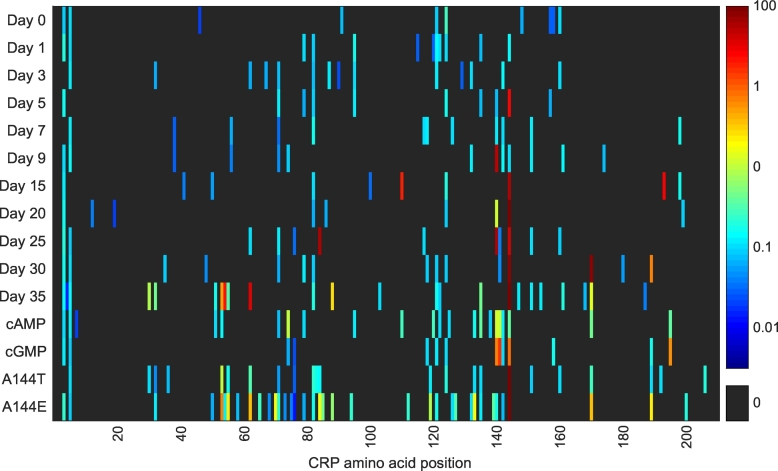
Heatmap of the mutation frequencies (%) of each CRP residue in an *E. coli* Δ*cyaA* pTIG strain. Strain and evolution experiment set-up were as previously described [48]. Biomass samples of the populations were taken from preculture (day 0) or from one of 20 identical plates during the evolution experiment (days 1–35). Furthermore, samples were taken at day 7 of the Δ*cyaA* strain supplemented with 0.1 mM cAMP or 0.5 mM cGMP, and of *E. coli* Δ*cyaA* strains with CRP A144T or CRP A144E mutations. The *crp* gene was sequenced by amplicon next-generation sequencing and the data processed by CLC Genomics Workbench (Qiagen, Aarhus, Denmark).

The new data presented here identifies more than 100 new *crp* missense mutations (Fig. 3, Fig. 4, Table S3), although we only observed a significant increase in different mutations towards the end of the 35 days. The HTS approach provides a previously unmatched look at the mutational landscape of CRP, revealing novel insights into the evolutionary response of a strain during a selective event. However, a tradeoff for the sequencing depth is its inability to distinguish between amplification of a single mutation and multiple occurrences of the same mutation. Thus, mutations detected by this HTS approach can only be roughly categorized into those appearing in higher frequencies (likely due to a clear fitness advantage leading to a dominating population) or those that appear in low frequencies. Similar, we cannot distinguish between mutations that occur alone and those that only occur in combination with other mutations such as described above for the mutation Q170K.

The data presented in Figs. 3 and 4 is generally well aligned with previous observations. Several high-frequency mutations are observed in the residues D53, S62, T140, G141, A144, Q170, and relatively few mutations are seen in the C-terminus of CRP. In addition, DNA was isolated from aging bacteria in the presence of two CRP ligands: 0.1 mM cAMP and 0.5 mM cGMP, and with parental strains that already contained the frequently observed CRP* mutants A144T and A144E (Fig. 4). From plates supplemented with the two cyclic nucleotides, binding site mutations again dominate together with mutations in the D-α-helix. Interestingly, although the A144T/E mutations observed by Sekowska *et al*. occur in the same residue, they have distinct effects on the mutational landscape of CRP. The A144T mutation causes almost uniformly distributed low frequency mutations, while the A144E mutation causes increased mutagenesis generally concentrated around residues 50–90 (AR3 and the cAMP binding site) and 110–150 (the cAMP binding site and inter-domain stability).

To our knowledge, the data presented here is the most comprehensive overview of the natural mutational landscape of CRP to date. Our approach provides a detailed map of CRP mutations for future in-depth characterization. With the increasingly affordable deep sequencing methodologies, the approach could be generalized to study similar evolutionary tracks in transcriptional regulation. How can we try and extend our knowledge of structural and regulatory features of the CRP protein?

Besides exploring changes in physical growth conditions (altering temperature or osmolarity) coupling with other master regulatory systems should be rewarding. Global regulators such as CRP manage coordination of gene expression in a variety of conditions, that are also coordinated by other regulatory molecules, in particular the alarmone ppGpp. This “magic spot” has been discovered half a century ago as involved in monitoring amino acid availability [8]. Yet its role is far from fully understood and still a matter of considerable research. It is now known that altered levels of this regulatory molecule in *relA spoT* mutants – coding for enzymes controlling the synthesis and turnover of the molecule – resulted in non-optimal resource allocation in *E. coli* [68]. Interestingly, this happened under conditions where it is expected that CRP is involved in the management of ppGpp-mediated effects [49] and CRP-mediated contribution to *relA* expression has been demonstrated [34]. This regulation must match the coupling between the cAMP-CRP regulation and amino acid biosynthesis.

As a case in point indeed, it has long been known that there is an explicit link between ppGpp synthesis and serine/one carbon metabolism. Serine excess resulted in growth inhibition of *relA* mutants, while *relA cya*-defective or *crp*-defective mutants became resistant to excess serine. In *relA cya* strains, sensitivity to serine was restored when the growth medium was supplemented with cAMP, substantiating the serine-mediated interference in the cAMP-CRP control of gene expression [10]. To be sure, this effect was reverted in a crp* background. It was therefore interesting to isolate secondary mutants that would again be resistant to excess serine in order to better understand how CRP was involved in this regulation. A new class of CRP mutants was identified in E. coli cya relA crp* strains. These mutants were mapped in the *crp* gene, and their physiological features differed from both the wild type *crp* and the crp* allele [11]. However, they could not be studied more in-depth at the time. Exploring this selection procedure with the “omics” techniques that are now familiar should allow us to enter a new evolution landscape of the protein.

Similar approaches could be developed to study other global regulators. In general, letting genes that are expressed under stationary conditions evolve should bring about new observations in the unchartered territory of adaptive mutations.

## Acknowledgements

We would like to thank the three anonymous reviewers for their positive, insightful and constructive comments. This work was supported by the Novo Nordisk Foundation.

## Appendix A Supplementary data

Supplementary material Image 1
