# Supplementary material for: Mutations in the Global Transcription Factor CRP/CAP: Insights from Experimental Evolution and Deep Sequencing
Source: Comput Struct Biotechnol J. 2019 Jun 18;17:730–6. doi: 10.1016/j.csbj.2019.05.009 (PMC6603298; doi:10.1016/j.csbj.2019.05.009)
Supplement: Supplementary file 1 — Supplementary material [file mmc1.docx]

**Supplementary information**

**Materials and Methods**

*Bacterial strains.* The strains applied in this study were *Escherichia coli* K12 MG1655 *ΔcyaA::cat* strains carrying either WT CRP and the pTrc*tig* plasmid (expressing the Tig chaperone), the CRP A144T mutation or the CRP A144E mutation (Sekowska et al., 2016). The strains were cultivated at 37°C in either LB medium (routine experiments) or MacConkey (Macconkey, 1905) medium with 1% maltose (evolution experiments) supplemented with appropriate antibiotics.

*Adaptive evolution experiment.* The strains in question were streaked on LB plates, from which single colonies were used to inoculate precultures (5 mL LB) for the adaptive evolution experiment. Precultures were grown for exactly 7 hours before being diluted to OD_600_=1 in LB and then to OD_600_=10^-4^ in 0.9% NaCl to ensure equal nutrients present in all samples. From the dilutions, 100 µL were plated on MacConkey maltose plates, supplemented if necessary with 0.1 mM cAMP or 0.5 mM cGMP, and the plates were incubated in a box with water to ensure constant humidity throughout the experiment.

*Biomass samples.* For the time series of the WT CRP background, biomass samples of the populations were taken from preculture (day 0) or from one of 20 identical plates of the adaptive evolution experiment. Furthermore, biomass samples were taken at day 7 of the WT CRP background supplemented with cAMP or cGMP, as well as of the CRP A144T and CRP A144E backgrounds, using 10 identical plates for each condition. The biomass samples were obtained by scraping off all biomass of plates using liquid LB medium and a drigalski spatula. The dissolved biomass was centrifuged and the pellet saved at -80°C. Genomic DNA was purified from the biomass samples using the DNeasy Blood & Tissue Kit (Qiagen).

*Amplicon next-generation sequencing of crp.* Three amplicons of 293, 282 and 338 bp, respectively, were designed to cover the entire *crp* gene, and were amplified using the adapter oligonucleotides shown in Table X. The amplicons were cleaned up using the NucleoSpin Gel and PCR Clean-up Kit (Macherey-Nagel) and eluted in 10 mM Tris-HCl pH 8.5. The library preparation was completed using the KAPA HyperPlus Kit (Roche), and the amplicons were sequenced on the MiSeq system (Illumina) using the MiSeq V3 Reagent Kit with 600 cycles (Illumina). The data was demultiplexed in the system before download.

*Processing of amplicon sequencing data.* The data processing was completed using CLC Genomics Workbench (CLC bio). FastQ files were imported into the workbench as paired reads. The paired reads were then merged by overlapping regions, and non-mergeable reads were discarded. The reads were them trimmed by quality scores (discarding those with p>0.001), ambiguous nucleotides (not allowed), adapters (forward and reverse complement, in case of amplification errors) and length (based on the minimum amplicon length without adapters). Trimmed reads were then mapped to a *crp* reference sequence using global alignment with standard alignment settings (match score = 1, mismatch cost = 2, linear gap cost = 3, length fraction = 0.5, similarity fraction = 0.8). Following this, the reads were examined for structural variants (quality p < 0.0001 and a maximum number of mismatches = 3 for end breakpoints), and this information was applied on the mapping for local realignment (two iterations, maximum variant length = 200 bp). Nucleotide variants were called and annotated by the software using low-frequency variant detection (no minimum variant count, neighbourhood quality filter = p<0.0003 in a 5 base radius, no set ploidy).

The adapter oligonucleotides applied for amplicon generation. Bold = *crp* annealing.

| Amplicon | Length (bp) | Oligonucleotides |
| --- | --- | --- |
| 1 | 293 | TCGTCGGCAGCGTCAGATGTGTATAAGAGACAG**CGTTATCTGGCTCTGGAGAAA** |
|  |  | GTCTCGTGGGCTCGGAGATGTGTATAAGAGACAG**GTTCCTGGCCCTCTTCAAAC** |
| 2 | 282 | TCGTCGGCAGCGTCAGATGTGTATAAGAGACAG**GGGTGATTTTATTGGCGAAC** |
|  |  | GTCTCGTGGGCTCGGAGATGTGTATAAGAGACAG**GGGTGAGTCATAGCGTCTGG** |
| 3 | 338 | TCGTCGGCAGCGTCAGATGTGTATAAGAGACAG**AGAGAAAGTGGGCAACCTG** |
|  |  | GTCTCGTGGGCTCGGAGATGTGTATAAGAGACAG**GCATAGTTGATATCGGGGTGA** |

**Table S1.** *In vivo* selected CRP mutants.

| Position | Mutation | Additional mutation(s) | Strain genotype (+ or Δ for *cya, crp)* | *crp* location | Type of mutagenesis | | Selection carbon source | Reference(s) |
| --- | --- | --- | --- | --- | --- | --- | --- | --- |
| L11 | I | A144T | *Δcya, crp+* | Genome | |  | Maltose | (Sekowska et al., 2016) |
| H17 | R |  | *Δcya, Δcrp* | Plasmid | |  |  | (Søgaard-Andersen et al., 1991) |
| C18 | R |  | *Δcya, Δcrp* | Plasmid | |  |  | (Søgaard-Andersen et al., 1991) |
| H19 | L, Y |  | *cya+, crpΔ* | Plasmid | | PCR | Lactose | (Niu et al., 1996) |
| H21 | L |  | *cya+, crpΔ* | Plasmid | | PCR | Lactose | (Niu et al., 1996) |
| T28 | K | A144T,E | *Δcya, crp+* | Genome | |  | Lactose | (Tagami et al., 1995) |
| I30 | F | A144T,E | *Δcya, crp+* | Genome | |  | Maltose | (Sekowska et al., 2016) |
| L39 | M | A144T,E | *Δcya, crp+* | Genome | |  | Maltose | (Sekowska et al., 2016) |
| D53 | H |  | *Δcya, crp+* | Plasmid | | UV | Lactose | (Aiba et al., 1985) |
|  | N | A145T | *Δcya, crp+* | Genome | |  | Maltose | (Sekowska et al., 2016) |
| E55 | D | A144T | *Δcya, crp+* | Genome | |  | Maltose | (Sekowska et al., 2016) |
|  | E | A144T | *Δcya, crp+* | Genome | |  | Maltose | (Sekowska et al., 2016) |
|  | K | A144T,E | *Δcya, crp+* | Genome | |  | Maltose | (Sekowska et al., 2016) |
| S62 | F |  | *Δcya, crp+* | Plasmid | | UV | Lactose | (Aiba et al., 1985) |
|  | F | A144T | *Δcya, crp+* | Genome | |  | Maltose | (Sekowska et al., 2016) |
|  | Y |  | *Δcya, crp+* | Genome | |  | Maltose | (Sekowska et al., 2016) |
|  | Y | A144T,E | *Δcya, crp+* | Genome | |  | Maltose | (Sekowska et al., 2016) |
| Y63 | F | M189K | *Δcya, crp+* | Genome | |  | Maltose | (Sekowska et al., 2016) |
| L64 | Q | A144T | *Δcya, crp+* | Genome | |  | Maltose | (Sekowska et al., 2016) |
| E72 | A |  | *Δcya, crp+* | Plasmid | | UV | Lactose | (Garges and Adhya, 1985) |
|  | V |  | *cya+, crpΔ* | Plasmid | | UV | Lactose+Galactose | (Eschenlauer and Reznikoff, 1991) |
| R82 | S | A144T | *Δcya, crp+* | Genome | |  | Maltose | (Sekowska et al., 2016) |
| A84 | E | A144T,E | *Δcya, crp+* | Genome | |  | Maltose | (Sekowska et al., 2016) |
| W85 | R | A144T | *Δcya, crp+* | Genome | |  | Maltose | (Sekowska et al., 2016) |
| K101 | E |  | *cya+, crpΔ* | Plasmid | | PCR | Lactose | (Niu et al., 1996) |
| V108 | A |  | *Δcya, Δcrp* | Plasmid | |  |  | (Søgaard-Andersen et al., 1991) |
| P110 | Q |  | *Δcya, crp+* | Genome | |  | Maltose | (Sekowska et al., 2016) |
|  | S |  | *Δcya, Δcrp* | Plasmid | |  |  | (Søgaard-Andersen et al., 1991) |
|  | Q | A144T | *Δcya, crp+* | Genome | |  | Maltose | (Sekowska et al., 2016) |
| Q119 | H | T140R, A144E | *Δcya, crp+* | Genome | |  | Maltose | (Sekowska et al., 2016) |
| T127 | A | G141S | *Δcya, Δcrp* | Plasmid | | UV | Lactose | (Garges and Adhya, 1988) |
|  | I | A144T | *Δcya, crp+* | Genome | |  | Maltose | (Sekowska et al., 2016) |
|  | I | Q170K | *Δcya, crp+* | Genome | | Chemical + UV | Lactose | (Harman et al., 1986; Puskas et al., 1983; Sanders and McGeoch, 1973) |
|  | I | Q170K + L195R | *Δcya, crp+* | Genome | | Chemical + UV | Arabinose | (Harman et al., 1986; Puskas et al., 1983) |
| N133 | H | A144T | *Δcya, crp+* | Genome | |  | Maltose | (Sekowska et al., 2016) |
| L134 | M |  | *Δcya, crp+* | Genome | |  | Maltose | (Sekowska et al., 2016) |
| T140 | P, R, K |  | *Δcya, crp+* | Genome | |  | Maltose | (Sekowska et al., 2016) |
|  | K |  | *Δcya, crp+* | Plasmid | |  | Lactose | (Tagami et al., 1995) |
|  | R | Q119H, Q170K, V183A | *Δcya, crp+* | Genome | |  | Maltose | (Sekowska et al., 2016) |
|  | K | A144T,E, M198R | *Δcya, crp+* | Genome | |  | Maltose | (Sekowska et al., 2016) |
| G141 | D |  | *Δcya, crp+* | Plasmid | | UV | Lactose | (Aiba et al., 1985) |
|  | D |  | *Δcya, crp+* | Plasmid | |  | Lactose | (Tagami et al., 1995) |
|  | D |  | *cya+, crp+* | Genome | |  | Xylose | (Sievert et al., 2017) |
|  | D,S |  | *Δcya, crp+* | Genome | |  | Maltose | (Sekowska et al., 2016) |
|  | S |  | *Δcya, crp+* | Plasmid | | UV | Lactose | (Garges and Adhya, 1985) |
|  | S | T127A | *Δcya, Δcrp* | Plasmid | | UV | Lactose | (Garges and Adhya, 1988) |
| R142 | C |  | *Δcya, crp+* | Plasmid | | UV | Lactose | (Aiba et al., 1985) |
|  | H |  | *Δcya, crp+* | Plasmid | | UV | Lactose | (Garges and Adhya, 1985) |
| A144 | T |  | *Δcya, crp+* | Plasmid | | UV | Lactose | (Garges and Adhya, 1985) |
|  | T |  | *Δcya, crp+* | Genome | |  | Maltose | (Sekowska et al., 2016) |
|  | T |  | *Δcya, crp+* | Genome | | Chemical | Xylose | (Harman et al., 1986; Melton et al., 1981) |
|  | T | T28K | *Δcya, crp+* | Genome | |  | Lactose | (Tagami et al., 1995) |
|  | T | R169C + E171G | *Δcya, Δcrp* | Plasmid | | UV | Lactose | (Garges and Adhya, 1988) |
|  | T | L11I, I30F, L39M, D53N, E55D,K,E, S62F,Y, L64Q, R82S, A84E, W85R, P110Q, T127I, N133H, T140K, Q145K, T146A, D155N, Q170K, M189I, M189K, Q193K | *Δcya, crp+* | Genome | |  | Maltose | (Sekowska et al., 2016) |
|  | E, K |  | *Δcya, crp+* | Genome | |  | Maltose | (Sekowska et al., 2016) |
|  | E | T28K | *Δcya, crp+* | Genome | |  | Lactose | (Tagami et al., 1995) |
|  | E | I30F, L39M, E55K, S62Y, A84E, Q119H, T140K, Q170K, Q174K, M189K | *Δcya, crp+* | Genome | |  | Maltose | (Sekowska et al., 2016) |
| Q145 | K | A144T | *Δcya, crp+* | Genome | |  | Maltose | (Sekowska et al., 2016) |
| T146 | A | A144T | *Δcya, crp+* | Genome | |  | Maltose | (Sekowska et al., 2016) |
| L148 | R |  | *Δcya, crp+* | Plasmid | | UV | Lactose | (Aiba et al., 1985) |
| L150 | Q |  | *cya+, crp+* | Genome | |  | Lactate | (Conrad et al., 2009) |
| D155 | N | A144T | *Δcya, crp+* | Genome | |  | Maltose | (Sekowska et al., 2016) |
| A156 | D |  | *cya+, crpΔ* | Plasmid | | PCR | Lactose+Ribose | (Zhou et al., 1993) |
| T158 | A, I |  | *cya+, crpΔ* | Plasmid | | PCR | Lactose+Ribose; Lactose | (Niu et al., 1996; Zhou et al., 1993) |
| H159 | L |  | *cya+, crpΔ* | Plasmid | | PCR | Lactose+Ribose | (Zhou et al., 1993) |
|  | R |  | *cya+, crpΔ* | Plasmid | | PCR | Lactose+Ribose; Lactose | (Niu et al., 1996; Zhou et al., 1993) |
| P160 | T |  | *cya+, crpΔ* | Plasmid | | PCR | Lactose | (Niu et al., 1996) |
| G162 | D |  | *cya+, crpΔ* | Plasmid | | PCR | Lactose+Ribose; Lactose | (Niu et al., 1996; Zhou et al., 1993) |
|  | C,N |  | *cya+, crpΔ* | Plasmid | | UV | Lactose+Galactose | (Eschenlauer and Reznikoff, 1991) |
|  | S |  | *cya+, crpΔ* | Plasmid | | PCR | Lactose+Ribose | (Zhou et al., 1993) |
|  | V, R |  | *cya+, crpΔ* | Plasmid | | PCR | Lactose | (Niu et al., 1996) |
| I165 | T |  | *cya+, crp+* | Genome | |  | Lactate | (Conrad et al., 2009) |
| R169 | C | A144T + E171G | *Δcya, crp+* | Plasmid | | UV | Lactose | (Garges and Adhya, 1988) |
| Q170 | K | T140R, A144T,E, M198K | *Δcya, crp+* | Genome | |  | Maltose | (Sekowska et al., 2016) |
|  | K | T127I | *Δcya, crp+* | Genome | | Chemical + UV | Lactose | (Harman et al., 1986; Puskas et al., 1983; Sanders and McGeoch, 1973) |
|  | K | T127I + L195R | *Δcya, crp+* | Genome | | Chemical + UV | Arabinose | (Harman et al., 1986; Puskas et al., 1983) |
| E171 | G | A144T + R169C | *Δcya, crp+* | Plasmid | | UV | Lactose | (Garges and Adhya, 1988) |
| Q174 | K | A145E | *Δcya, crp+* | Genome | |  | Maltose | (Sekowska et al., 2016) |
| V183 | A | T140R | *Δcya, crp+* | Genome | |  | Maltose | (Sekowska et al., 2016) |
| M189 | K |  | *Δcya, crp+* | Genome | |  | Maltose | (Sekowska et al., 2016) |
|  | K | Y63F, T140R, A144T,E, Q170K | *Δcya, crp+* | Genome | |  | Maltose | (Sekowska et al., 2016) |
|  | I | A144T | *Δcya, crp+* | Genome | |  | Maltose | (Sekowska et al., 2016) |
|  | R | T140K | *Δcya, crp+* | Genome | |  | Maltose | (Sekowska et al., 2016) |
| Q193 | K | A144T | *Δcya, crp+* | Genome | |  | Maltose | (Sekowska et al., 2016) |
| L195 | R |  | *Δcya, crp+* | Genome | |  | Maltose | (Sekowska et al., 2016) |
|  | R | T127I + Q170K | *Δcya, crp+* | Genome | | Chemical + UV | Arabinose | (Harman et al., 1986; Puskas et al., 1983) |

**Table S2.** Rationally engineered CRP mutants.

| Position | Mutation | Additional mutation(s) | CRP function | Rationale behind mutagenesis | Reference(s) |
| --- | --- | --- | --- | --- | --- |
| D8 | K |  |  | Transcriptional activation mechanism | (Zhang et al., 1992) |
| E12 | K |  |  | Transcriptional activation mechanism | (Zhang et al., 1992) |
| H19 | Y |  | AR2 | Polymerase interaction | (Rhodius et al., 1997) |
|  | Y | K101E | AR2 | Polymerase interaction | (Rhodius et al., 1997) |
| E34 | K |  |  | Transcriptional activation mechanism | (Zhang et al., 1992) |
| E37 | K |  |  | Transcriptional activation mechanism | (Zhang et al., 1992) |
| K52 | A |  |  | Polymerase interaction | (Rhodius and Busby, 2000) |
|  | N |  | AR3 | DNA binding; Ligand binding | (Bell et al., 1990; Dai et al., 2004; Lin et al., 2002; Williams et al., 1996) |
|  | N | H159L | AR3 | Ligand binding; Polymerase interaction | (Lin et al., 2002; West et al., 1993; Williams et al., 1996) |
|  | N | E96G | AR3 | Polymerase interaction | (Williams et al., 1996) |
|  | N | E96G + H159L | AR3 | Polymerase interaction | (Williams et al., 1996) |
|  | N | H159L + E181V | AR3 | Polymerase interaction | (Williams et al., 1996) |
|  | N | K101E + H159L | AR3 | Polymerase interaction | (Rhodius and Busby, 2000) |
|  | N | E58K + K101E + H159L | AR3 | Polymerase interaction | (Rhodius and Busby, 2000) |
|  | N | E58G + K101E + H159L | AR3 | Polymerase interaction | (Rhodius and Busby, 2000) |
|  | Q |  | AR3 | DNA binding | (Bell et al., 1990) |
|  | E | H159L | AR3 | Polymerase interaction | (West et al., 1993) |
| D53 | H |  | AR3 | Allosteric change; Ligand binding | (Dai et al., 2004; Lin et al., 2002; Tzeng and Kalodimos, 2009) |
|  | K |  | AR3 | Transcriptional activation mechanism | (Zhang et al., 1992) |
| E54 | K |  | AR3 | Transcriptional activation mechanism | (Zhang et al., 1992) |
| E55 | K |  | AR3 | Transcriptional activation mechanism | (Irwin and Ptashne, 1987; Zhang et al., 1992) |
| E58 | H |  | AR3, cAMP binding (*syn*) | Polymerase interaction | (West et al., 1993) |
|  | K |  | AR3, cAMP binding (*syn*) | Transcriptional activation mechanism; Polymerase interaction | (Rhodius and Busby, 2000; Zhang et al., 1992) |
|  | K | K52N + K101E + H159L | AR3, cAMP binding (*syn*) | Polymerase interaction | (Rhodius and Busby, 2000) |
|  | G |  | AR3, cAMP binding (*syn*) | Polymerase interaction | (Rhodius and Busby, 2000) |
|  | G | K52N + K101E + H159L | AR3, cAMP binding (*syn*) | Polymerase interaction | (Rhodius and Busby, 2000) |
| S62 | A |  |  | Crystal structure | (Gronenborn et al., 1988) |
|  | F |  |  | Allosteric change; Ligand binding | (Lin et al., 2002; Tzeng and Kalodimos, 2009) |
| D68 | K |  |  | Transcriptional activation mechanism | (Zhang et al., 1992) |
| E72 | D, Q |  | cAMP binding (*anti*) | Ligand binding | (Belduz et al., 1993; Moore et al., 1992) |
|  | D, Q | A144T | cAMP binding (*anti*) | Ligand binding | (Belduz et al., 1993; Moore et al., 1992) |
|  | A, P,G,H,R |  | cAMP binding (*anti*) | Ligand binding | (Moore et al., 1992) |
|  | A, P,G,H,R | A144T | cAMP binding (*anti*) | Ligand binding | (Moore et al., 1992) |
|  | L |  | cAMP binding (*anti*) | Ligand binding | (Belduz et al., 1993) |
|  | L | A144T | cAMP binding (*anti*) | Ligand binding | (Belduz et al., 1993) |
| E77 | K |  |  | Transcriptional activation mechanism | (Zhang et al., 1992) |
| E78 | K |  |  | Transcriptional activation mechanism | (Zhang et al., 1992) |
| E81 | K |  |  | Transcriptional activation mechanism | (Zhang et al., 1992) |
| R82 | K |  | cAMP binding (*anti*) | Ligand binding | (Belduz et al., 1993; Zhang et al., 1992) |
|  | K | A144T | cAMP binding (*anti*) | Ligand binding | (Belduz et al., 1993; Zhang et al., 1992) |
|  | A,T,E |  | cAMP binding (*anti*) | Ligand binding | (Moore et al., 1992) |
|  | A,T,E | A144T | cAMP binding (*anti*) | Ligand binding | (Moore et al., 1992) |
|  | L,Q,H |  | cAMP binding (*anti*) | Ligand binding | (Belduz et al., 1993) |
|  | L,Q,H | A144T | cAMP binding (*anti*) | Ligand binding | (Belduz et al., 1993) |
| S83 | A |  | cAMP binding (*anti*) | Crystal struture, Ligand binding | (Gronenborn et al., 1988; Moore et al., 1992) |
|  | A | A144T | cAMP binding (*anti*) | Ligand binding | (Moore et al., 1992) |
|  | K |  | cAMP binding (*anti*) | Crystal struture | (Gronenborn et al., 1988) |
|  | G,C |  | cAMP binding (*anti*) | Ligand binding | (Lee et al., 1994) |
|  | T,I,V |  | cAMP binding (*anti*) | Ligand binding | (Moore et al., 1992) |
|  | T,I,V | A144T | cAMP binding (*anti*) | Ligand binding | (Moore et al., 1992) |
| E93 | K |  |  | Transcriptional activation mechanism | (Zhang et al., 1992) |
| E96 | K |  | AR2 | Transcriptional activation mechanism | (Zhang et al., 1992) |
|  | G |  | AR2 | Polymerase interaction | (Williams et al., 1996) |
|  | G | K52N | AR2 | Polymerase interaction | (Williams et al., 1996) |
|  | G | K52N + H159L | AR2 | Polymerase interaction | (Williams et al., 1996) |
|  | G | H159L + E181V | AR2 | Polymerase interaction | (Williams et al., 1996) |
|  | G | H159L | AR2 | Polymerase interaction | (West et al., 1993; Williams et al., 1996) |
| K101 | E |  | AR2 | Polymerase interaction | (Rhodius et al., 1997; Rhodius and Busby, 2000) |
|  | E | H19Y | AR2 | Polymerase interaction | (Latif et al., 2018; Rhodius et al., 1997) |
|  | E | H159L | AR2 | Polymerase interaction | (Latif et al., 2018; Rhodius and Busby, 2000) |
|  | E | K52N + H159L | AR2 | Polymerase interaction | (Rhodius and Busby, 2000) |
|  | E | K52N + E58K + H159L | AR2 | Polymerase interaction | (Rhodius and Busby, 2000) |
|  | E | K52N + E58G + H159L | AR2 | Polymerase interaction | (Rhodius and Busby, 2000) |
| D111 | K |  |  | Transcriptional activation mechanism | (Zhang et al., 1992) |
| R123 | A,Q,E,P |  |  | Ligand binding | (Moore et al., 1992) |
|  | A,Q,E,P | A144T |  | Ligand binding | (Moore et al., 1992) |
| T127 | A |  | cAMP binding (*anti*) | Crystial structure, Ligand binding | (Gronenborn et al., 1988; Gunasekara et al., 2015) |
|  | A | S128V | cAMP binding (*anti*) | Ligand binding | (Gunasekara et al., 2015) |
|  | G |  | cAMP binding (*anti*) | Ligand binding | (Lee et al., 1994) |
|  | L |  | cAMP binding (*anti*) | Ligand binding | (Dai et al., 2004; Gorshkova et al., 1995; Lin et al., 2002; Wang et al., 2000) |
|  | L | S128I,V,M,L,A | cAMP binding (*anti*) | Ligand binding | (Youn et al., 2006) |
|  | L | S128L + L61V | cAMP binding (*anti*) | Ligand binding | (Youn et al., 2006) |
|  | L | S128I + 161V | cAMP binding (*anti*) | Ligand binding | (Youn et al., 2006) |
|  | L | S128N | cAMP binding (*anti*) | Ligand binding | (Gunasekara et al., 2015) |
|  | I |  | cAMP binding (*anti*) | Ligand binding | (Lee et al., 1994) |
|  | I | S128I | cAMP binding (*anti*) | Ligand binding | (Youn et al., 2006) |
|  | M |  | cAMP binding (*anti*) | Ligand binding | (Gunasekara et al., 2015) |
|  | M | S128I | cAMP binding (*anti*) | Ligand binding | (Youn et al., 2006) |
|  | V | S128I | cAMP binding (*anti*) | Ligand binding | (Youn et al., 2006) |
|  | V | S128T,V, A | cAMP binding (*anti*) | Ligand binding | (Gunasekara et al., 2015) |
|  | C | S128I | cAMP binding (*anti*) | Ligand binding | (Gunasekara et al., 2015) |
|  | C,S |  | cAMP binding (*anti*) | Ligand binding | (Gunasekara et al., 2015; Lee et al., 1994) |
|  | T | S128N | cAMP binding (*anti*) | Ligand binding | (Gunasekara et al., 2015) |
| S128 | A |  | cAMP binding (*anti*) | Crystal stucture, Ligand binding | (Cheng et al., 1995; Cheng and Ching Lee, 1998; Gronenborn et al., 1988; Lee et al., 1994; Moore et al., 1996; Wang et al., 2000) |
|  | A | G141Q | cAMP binding (*anti*) | Ligand binding | (Cheng and Ching Lee, 1998) |
|  | A | T127V |  | Ligand binding | (Gunasekara et al., 2015) |
|  | T |  | cAMP binding (*anti*) | Ligand binding | (Gunasekara et al., 2015; Lee et al., 1994) |
|  | P |  | cAMP binding (*anti*) | Ligand binding | (Cheng et al., 1995) |
|  | N |  | cAMP binding (*anti*) | Ligand binding | (Gunasekara et al., 2015) |
|  | N | T127L |  | Ligand binding | (Youn et al., 2006) |
|  | V | T127A |  | Ligand binding | (Youn et al., 2006) |
|  | V | T127V |  | Ligand binding | (Youn et al., 2006) |
|  | I | T127L,I,M,V,C |  | Ligand binding | (Youn et al., 2006) |
|  | I | T127L + L61V |  | Ligand binding | (Youn et al., 2006) |
|  | L | T127L + L61V |  | Ligand binding | (Youn et al., 2006) |
|  | T | T127V |  | Ligand binding | (Youn et al., 2006) |
| E129 | K |  |  | Transcriptional activation mechanism | (Zhang et al., 1992) |
| D138 | K |  |  | Transcriptional activation mechanism | (Zhang et al., 1992) |
|  | A,N,E,Q, G,L,K,F,V |  |  | Allosteric change | (Ryu et al., 1993) |
| G141 | Q |  |  | Ligand binding, Allosteric change | (Cheng and Ching Lee, 1998; Cheng and Lee, 1994; Dai et al., 2004; Kim et al., 1992; Lin et al., 2002) |
|  | S |  |  | Allosteric change | (Tzeng and Kalodimos, 2009) |
|  | S,Y,L,R,K,A,I,V |  |  | Allosteric change | (Kim et al., 1992) |
|  | K |  |  | Ligand binding | (Lin et al., 2002) |
| R142 | H | A144T |  | Allosteric change | (Tzeng and Kalodimos, 2009) |
| A144 | T |  |  | Ligand binding | (Belduz et al., 1993; Moore et al., 1992) |
|  | S,Q,Y,L,F,V,C |  |  | Allosteric change, CRP* phenotype | (Kim et al., 1992) |
|  | T | E72,D,Q,A,P,G,H,R, R82,K,A,T,E,L,Q,H, S83A,T,I,V, R123A,Q,E,P |  | Ligand binding, Allosteric change | (Belduz et al., 1993; Moore et al., 1992; Tzeng and Kalodimos, 2009) |
| L148 | R |  |  | Allosteric change; Ligand binding | (Lin et al., 2002; Tzeng and Kalodimos, 2009) |
| D155 | K |  |  | Transcriptional activation mechanism | (Zhang et al., 1992) |
| T158 | A |  | AR1 | Polymerase interaction | (West et al., 1993) |
| H159 | L |  | AR1 | Polymerase interaction | (Bell et al., 1990; Dai et al., 2004; Latif et al., 2018; Lin et al., 2002; Rhodius and Busby, 2000; West et al., 1993; Williams et al., 1996) |
|  | L | K101E | AR1 | Polymerase interaction | (Latif et al., 2018; Rhodius and Busby, 2000) |
|  | L | K52N | AR1 | Ligand binding; Polymerase interaction | (Lin et al., 2002; West et al., 1993; Williams et al., 1996) |
|  | L | K52E | AR1 | Polymerase interaction | (West et al., 1993) |
|  | L | E96G | AR1 | Polymerase interaction | (West et al., 1993; Williams et al., 1996) |
|  | L | K52N + K101E | AR1 | Polymerase interaction | (Rhodius and Busby, 2000) |
|  | L | K52N + E96G | AR1 | Polymerase interaction | (Williams et al., 1996) |
|  | L | K52N + E181V | AR1 | Polymerase interaction | (Williams et al., 1996) |
|  | L | E96G + E181V | AR1 | Polymerase interaction | (Williams et al., 1996) |
|  | L | K52N + E58K + K101E | AR1 | Polymerase interaction | (Rhodius and Busby, 2000) |
|  | L | K52N + E58G + K101E | AR1 | Polymerase interaction | (Rhodius and Busby, 2000) |
| D161 | K |  | AR1 | Transcriptional activation mechanism | (Zhang et al., 1992) |
| Q170 | K, E |  |  | Transcriptional activation mechanism | (Breul et al., 1993; Irwin and Ptashne, 1987) |
|  | L | E171K |  | Transcriptional activation mechanism | (Breul et al., 1993) |
| E171 | L |  |  | Transcriptional activation mechanism | (Breul et al., 1993) |
|  | K |  |  | Transcriptional activation mechanism | (Bell et al., 1990; Breul et al., 1993; Irwin and Ptashne, 1987; Zhang et al., 1992) |
|  | Q |  |  | Transcriptional activation mechanism | (Irwin and Ptashne, 1987) |
|  | K | Q170L |  | Transcriptional activation mechanism | (Breul et al., 1993) |
| R180 | A, G |  | cAMP binding *(syn*) | DNA binding | (Zhang and Ebright, 1990) |
| E181 | V |  |  | Polymerase interaction | (Williams et al., 1996) |
|  | V | K52N + H159L |  | Polymerase interaction | (Williams et al., 1996) |
|  | V | E96G + H159L |  | Polymerase interaction | (Williams et al., 1996) |
| E191 | K |  | DNA recognition | Transcriptional activation mechanism | (Irwin and Ptashne, 1987; Zhang et al., 1992) |
| D192 | K |  | DNA recognition | Transcriptional activation mechanism | (Zhang et al., 1992) |
| L195 | R |  |  | CRP* phenotype | (Harman et al., 1988) |

**Table S3.** The mutational landscape of *Escherichia coli* K12 MG1655 *ΔcyaA* populations during the adaptive evolution experiment. The *crp* genetic background is either wild-type (‘WT’, with the pTrc*tig* plasmid), CRP A144T (‘A144T’) or CRP A144E (‘A144E’). Age refers to the age of plates in the evolution experiment from which biomass samples were taken. Supplement refers to the nucleotide supplements to MacConkey maltose plates during the adaptive evolution experiment, where either no nucleotide (‘None’), 0.1 mM cAMP (‘cAMP’) or 0.5 mM cGMP (‘cGMP’) was supplemented. The frequency corresponds to the percentage of the population, which in the nucleotide position for each mutation contained the mutant variant as opposed to the wild-type variant.

| Amino acid |  | Mutation |  | *crp* genetic background | Age  (days) |  | Supplement |  | Frequency (%) |
| --- | --- | --- | --- | --- | --- | --- | --- | --- | --- |
| G3 |  | D |  | WT | 0 |  | None |  | 0.090 |
|  |  |  |  | WT | 1 |  | None |  | 0.098 |
|  |  |  |  | WT | 5 |  | None |  | 0.088 |
|  |  |  |  | WT | 9 |  | None |  | 0.108 |
|  |  |  |  | WT | 20 |  | None |  | 0.178 |
|  |  |  |  | WT | 25 |  | None |  | 0.209 |
|  |  |  |  | WT | 30 |  | None |  | 0.171 |
|  |  |  |  | WT | 35 |  | None |  | 0.148 |
|  |  |  |  | WT | 7 |  | cAMP |  | 0.097 |
|  |  |  |  | WT | 7 |  | cGMP |  | 0.127 |
|  |  |  |  | A144E | 7 |  | None |  | 0.094 |
|  |  |  |  | A144E | 7 |  | None |  | 0.017 |
|  |  | Silent |  | WT | 1 |  | None |  | 0.098 |
|  |  |  |  | WT | 5 |  | None |  | 0.088 |
|  |  |  |  | WT | 15 |  | None |  | 0.019 |
|  |  |  |  | WT | 15 |  | None |  | 0.103 |
|  |  |  |  | A144E | 7 |  | None |  | 0.017 |
|  |  |  |  | A144E | 7 |  | None |  | 0.094 |
| K4 |  | N |  | WT | 35 |  | None |  | 0.016 |
| P5 |  | A |  | WT | 7 |  | cAMP |  | 0.032 |
|  |  | Silent |  | WT | 0 |  | None |  | 0.110 |
|  |  |  |  | WT | 1 |  | None |  | 0.098 |
|  |  |  |  | WT | 3 |  | None |  | 0.121 |
|  |  |  |  | WT | 7 |  | None |  | 0.121 |
|  |  |  |  | WT | 9 |  | None |  | 0.144 |
|  |  |  |  | WT | 25 |  | None |  | 0.090 |
|  |  |  |  | WT | 30 |  | None |  | 0.106 |
|  |  |  |  | WT | 35 |  | None |  | 0.133 |
|  |  |  |  | WT | 7 |  | cAMP |  | 0.097 |
|  |  |  |  | WT | 7 |  | cGMP |  | 0.088 |
|  |  |  |  | A144T | 7 |  | None |  | 0.088 |
|  |  |  |  | A144T | 7 |  | None |  | 0.016 |
|  |  |  |  | A144E | 7 |  | None |  | 0.094 |
| T7 |  | Silent |  | WT | 7 |  | cAMP |  | 0.022 |
| E12 |  | * |  | WT | 20 |  | None |  | 0.042 |
| H19 |  | P |  | WT | 20 |  | None |  | 0.021 |
| I30 |  | F |  | WT | 35 |  | None |  | 0.859 |
|  |  |  |  | A144T | 7 |  | None |  | 0.096 |
| Q32 |  | K |  | WT | 3 |  | None |  | 0.060 |
|  |  | P |  | WT | 35 |  | None |  | 0.431 |
|  |  |  |  | A144T | 7 |  | None |  | 0.048 |
|  |  |  |  | A144E | 7 |  | None |  | 0.129 |
| K35 |  | Silent |  | WT | 30 |  | None |  | 0.082 |
| A36 |  | V |  | A144T | 7 |  | None |  | 0.080 |
| T38 |  | P |  | WT | 7 |  | None |  | 0.030 |
|  |  |  |  | WT | 9 |  | None |  | 0.027 |
| Y41 |  | F |  | WT | 15 |  | None |  | 0.037 |
| S46 |  | Silent |  | WT | 0 |  | None |  | 0.020 |
| A48 |  | V |  | WT | 30 |  | None |  | 0.049 |
| L50 |  | V |  | WT | 15 |  | None |  | 0.019 |
|  |  |  |  | WT | 15 |  | None |  | 0.038 |
|  |  | P |  | A144E | 7 |  | None |  | 0.060 |
| I51 |  | L |  | WT | 35 |  | None |  | 0.157 |
|  |  |  |  | WT | 7 |  | cAMP |  | 0.097 |
| D53 |  | N |  | WT | 35 |  | None |  | 3.003 |
|  |  |  |  | A144T | 7 |  | None |  | 0.500 |
|  |  |  |  | A144E | 7 |  | None |  | 4.667 |
|  |  | H |  | A144E | 7 |  | None |  | 0.129 |
|  |  | Y |  | WT | 35 |  | None |  | 0.141 |
|  |  |  |  | WT | 7 |  | cAMP |  | 0.140 |
|  |  |  |  | A144T | 7 |  | None |  | 0.193 |
|  |  |  |  | A144E | 7 |  | None |  | 1.380 |
|  |  | A |  | A144T | 7 |  | None |  | 0.089 |
|  |  |  |  | A144E | 7 |  | None |  | 0.087 |
|  |  | G |  | WT | 35 |  | None |  | 1.498 |
|  |  |  |  | A144T | 7 |  | None |  | 0.113 |
|  |  |  |  | A144E | 7 |  | None |  | 0.831 |
|  |  | V |  | A144T | 7 |  | None |  | 0.024 |
|  |  |  |  | A144E | 7 |  | None |  | 0.061 |
| E54 |  | K |  | WT | 35 |  | None |  | 21.107 |
|  |  |  |  | A144E | 7 |  | None |  | 0.120 |
| E55 |  | K |  | WT | 35 |  | None |  | 0.205 |
|  |  |  |  | A144T | 7 |  | None |  | 0.090 |
|  |  |  |  | A144E | 7 |  | None |  | 2.013 |
|  |  | G |  | WT | 35 |  | None |  | 0.120 |
|  |  |  |  | A144T | 7 |  | None |  | 0.082 |
|  |  |  |  | A144E | 7 |  | None |  | 0.290 |
| G56 |  | V |  | WT | 9 |  | None |  | 0.036 |
|  |  | Silent |  | WT | 7 |  | None |  | 0.063 |
| E58 |  | Q |  | A144E | 7 |  | None |  | 0.070 |
| S62 |  | F |  | WT | 25 |  | None |  | 0.100 |
|  |  |  |  | WT | 35 |  | None |  | 36.783 |
|  |  |  |  | A144T | 7 |  | None |  | 0.257 |
|  |  |  |  | A144E | 7 |  | None |  | 2.963 |
|  |  | Y |  | A144T | 7 |  | None |  | 0.088 |
|  |  |  |  | A144E | 7 |  | None |  | 0.629 |
|  |  | Silent |  | WT | 3 |  | None |  | 0.060 |
| N65 |  | H |  | A144E | 7 |  | None |  | 0.217 |
|  |  | Y |  | A144E | 7 |  | None |  | 0.063 |
| G67 |  | Silent |  | WT | 3 |  | None |  | 0.044 |
|  |  |  |  | WT | 3 |  | None |  | 0.018 |
| D68 |  | G |  | A144E | 7 |  | None |  | 0.009 |
|  |  |  |  | A144E | 7 |  | None |  | 0.026 |
|  |  | E |  | A144E | 7 |  | None |  | 0.009 |
| I70 |  | F |  | A144E | 7 |  | None |  | 1.557 |
|  |  | S |  | A144E | 7 |  | None |  | 0.026 |
|  |  | V |  | A144E | 7 |  | None |  | 0.036 |
|  |  | T |  | A144E | 7 |  | None |  | 0.174 |
| G71 |  | D |  | A144E | 7 |  | None |  | 0.036 |
|  |  | V |  | WT | 5 |  | None |  | 0.023 |
|  |  | Silent |  | WT | 3 |  | None |  | 0.072 |
|  |  |  |  | WT | 5 |  | None |  | 0.054 |
|  |  |  |  | WT | 5 |  | None |  | 0.054 |
|  |  |  |  | WT | 7 |  | None |  | 0.032 |
|  |  |  |  | WT | 9 |  | None |  | 0.047 |
|  |  |  |  | WT | 25 |  | None |  | 0.115 |
|  |  |  |  | WT | 30 |  | None |  | 0.068 |
|  |  |  |  | WT | 7 |  | cAMP |  | 0.079 |
|  |  |  |  | A144T | 7 |  | None |  | 0.050 |
|  |  |  |  | A144E | 7 |  | None |  | 0.054 |
|  |  |  |  | A144E | 7 |  | None |  | 0.054 |
| L73 |  | V |  | A144E | 7 |  | None |  | 0.044 |
| G74 |  | C |  | WT | 7 |  | cAMP |  | 1.070 |
|  |  | Silent |  | WT | 9 |  | None |  | 0.082 |
|  |  |  |  | WT | 7 |  | cGMP |  | 0.064 |
| L75 |  | Q |  | A144E | 7 |  | None |  | 0.040 |
| F76 |  | V |  | WT | 7 |  | cGMP |  | 0.022 |
|  |  | C |  | A144T | 7 |  | None |  | 0.029 |
|  |  | L |  | WT | 25 |  | None |  | 0.036 |
|  |  | Silent |  | A144E | 7 |  | None |  | 0.012 |
| G79 |  | A |  | A144E | 7 |  | None |  | 0.059 |
|  |  | Silent |  | WT | 1 |  | None |  | 0.109 |
|  |  |  |  | WT | 5 |  | None |  | 0.032 |
|  |  |  |  | WT | 5 |  | None |  | 0.032 |
|  |  |  |  | WT | 30 |  | None |  | 0.121 |
|  |  |  |  | WT | 7 |  | cAMP |  | 0.100 |
| R82 |  | G |  | WT | 35 |  | None |  | 0.205 |
|  |  |  |  | A144T | 7 |  | None |  | 0.117 |
|  |  |  |  | A144E | 7 |  | None |  | 0.180 |
|  |  | H |  | A144T | 7 |  | None |  | 0.043 |
|  |  | P |  | WT | 1 |  | None |  | 0.093 |
|  |  |  |  | WT | 3 |  | None |  | 0.077 |
|  |  |  |  | WT | 5 |  | None |  | 0.094 |
|  |  |  |  | WT | 7 |  | None |  | 0.159 |
|  |  |  |  | WT | 15 |  | None |  | 0.102 |
|  |  |  |  | WT | 20 |  | None |  | 0.061 |
|  |  |  |  | WT | 30 |  | None |  | 0.093 |
|  |  |  |  | A144T | 7 |  | None |  | 0.072 |
| S83 |  | G |  | A144T | 7 |  | None |  | 0.175 |
| A84 |  | E |  | WT | 25 |  | None |  | 54.226 |
|  |  |  |  | A144T | 7 |  | None |  | 0.135 |
|  |  |  |  | A144E | 7 |  | None |  | 1.514 |
| W85 |  | R |  | A144E | 7 |  | None |  | 0.441 |
| V86 |  | Silent |  | WT | 20 |  | None |  | 0.061 |
| R87 |  | H |  | WT | 3 |  | None |  | 0.116 |
| A88 |  | V |  | WT | 35 |  | None |  | 2.813 |
|  |  |  |  | A144E | 7 |  | None |  | 0.595 |
| T90 |  | P |  | WT | 3 |  | None |  | 0.026 |
| A91 |  | T |  | WT | 0 |  | None |  | 0.080 |
| V94 |  | L |  | A144E | 7 |  | None |  | 0.236 |
| A95 |  | P |  | WT | 1 |  | None |  | 0.027 |
|  |  | T |  | WT | 1 |  | None |  | 0.080 |
|  |  |  |  | WT | 5 |  | None |  | 0.126 |
|  |  | Silent |  | WT | 1 |  | None |  | 0.067 |
|  |  |  |  | WT | 3 |  | None |  | 0.090 |
|  |  |  |  | WT | 3 |  | None |  | 0.013 |
|  |  |  |  | WT | 7 |  | cAMP |  | 0.123 |
| K100 |  | Q |  | WT | 15 |  | None |  | 0.034 |
| R103 |  | H |  | WT | 35 |  | None |  | 0.103 |
| P110 |  | Q |  | WT | 15 |  | None |  | 18.110 |
|  |  |  |  | WT | 7 |  | cAMP |  | 0.270 |
| I112 |  | L |  | A144E | 7 |  | None |  | 0.249 |
| R115 |  | Silent |  | WT | 1 |  | None |  | 0.027 |
| S117 |  | Silent |  | WT | 7 |  | None |  | 0.100 |
|  |  |  |  | WT | 25 |  | None |  | 0.106 |
| A118 |  | T |  | WT | 30 |  | None |  | 0.093 |
|  |  |  |  | WT | 7 |  | cGMP |  | 0.109 |
|  |  | V |  | WT | 7 |  | None |  | 0.119 |
| Q119 |  | H |  | A144T | 7 |  | None |  | 0.116 |
|  |  |  |  | A144E | 7 |  | None |  | 0.177 |
|  |  |  |  | A144E | 7 |  | None |  | 0.734 |
| M120 |  | L |  | WT | 1 |  | None |  | 0.027 |
|  |  | I |  | WT | 7 |  | cAMP |  | 0.234 |
|  |  |  |  | WT | 7 |  | cAMP |  | 0.037 |
| A121 |  | E |  | A144E | 7 |  | None |  | 0.047 |
|  |  | G |  | WT | 7 |  | cGMP |  | 0.022 |
|  |  | V |  | WT | 0 |  | None |  | 0.091 |
|  |  |  |  | WT | 1 |  | None |  | 0.134 |
|  |  |  |  | WT | 3 |  | None |  | 0.116 |
|  |  |  |  | WT | 30 |  | None |  | 0.094 |
|  |  |  |  | WT | 35 |  | None |  | 0.176 |
|  |  |  |  | WT | 7 |  | cGMP |  | 0.098 |
|  |  |  |  | A144E | 7 |  | None |  | 0.131 |
| R122 |  | H |  | WT | 1 |  | None |  | 0.108 |
|  |  |  |  | WT | 35 |  | None |  | 0.088 |
|  |  |  |  | WT | 7 |  | cAMP |  | 0.099 |
| L124 |  | P |  | WT | 0 |  | None |  | 0.091 |
|  |  |  |  | WT | 15 |  | None |  | 0.086 |
|  |  |  |  | WT | 7 |  | cGMP |  | 0.109 |
|  |  | Silent |  | WT | 0 |  | None |  | 0.148 |
|  |  |  |  | WT | 0 |  | None |  | 0.023 |
|  |  |  |  | WT | 1 |  | None |  | 0.107 |
|  |  |  |  | WT | 5 |  | None |  | 0.173 |
|  |  |  |  | WT | 15 |  | None |  | 0.120 |
|  |  |  |  | WT | 20 |  | None |  | 0.092 |
|  |  |  |  | WT | 30 |  | None |  | 0.093 |
|  |  |  |  | A144T | 7 |  | None |  | 0.203 |
| Q125 |  | K |  | WT | 7 |  | cAMP |  | 0.098 |
| V126 |  | A |  | WT | 7 |  | None |  | 0.100 |
|  |  | D |  | A144E | 7 |  | None |  | 0.107 |
| T127 |  | I |  | A144E | 7 |  | None |  | 0.308 |
| E129 |  | A |  | WT | 3 |  | None |  | 0.026 |
| G132 |  | Silent |  | WT | 3 |  | None |  | 0.103 |
|  |  |  |  | WT | 9 |  | None |  | 0.116 |
|  |  |  |  | A144E | 7 |  | None |  | 0.012 |
|  |  |  |  | A144E | 7 |  | None |  | 0.083 |
| N133 |  | H |  | WT | 7 |  | cAMP |  | 0.111 |
|  |  |  |  | A144T | 7 |  | None |  | 0.102 |
|  |  |  |  | A144E | 7 |  | None |  | 1.222 |
|  |  | Y |  | WT | 7 |  | cAMP |  | 0.062 |
|  |  | Silent |  | A144E | 7 |  | None |  | 0.629 |
| A135 |  | V |  | WT | 5 |  | None |  | 0.073 |
|  |  |  |  | WT | 35 |  | None |  | 0.409 |
|  |  |  |  | WT | 7 |  | cAMP |  | 0.369 |
|  |  |  |  | A144T | 7 |  | None |  | 0.111 |
|  |  |  |  | A144E | 7 |  | None |  | 0.117 |
|  |  | Silent |  | WT | 1 |  | None |  | 0.077 |
| D138 |  | E |  | WT | 7 |  | cAMP |  | 0.093 |
| V139 |  | A |  | A144E | 7 |  | None |  | 0.374 |
| T140 |  | P |  | WT | 7 |  | cGMP |  | 1.083 |
|  |  | S |  | WT | 7 |  | cGMP |  | 0.018 |
|  |  | R |  | WT | 5 |  | None |  | 0.073 |
|  |  |  |  | WT | 7 |  | None |  | 0.051 |
|  |  |  |  | WT | 9 |  | None |  | 47.513 |
|  |  |  |  | WT | 25 |  | None |  | 53.574 |
|  |  |  |  | A144E | 7 |  | None |  | 0.180 |
|  |  | K |  | WT | 20 |  | None |  | 1.215 |
|  |  |  |  | WT | 7 |  | cAMP |  | 1.019 |
|  |  |  |  | WT | 7 |  | cGMP |  | 5.525 |
|  |  | M |  | WT | 7 |  | None |  | 0.076 |
|  |  |  |  | WT | 7 |  | cAMP |  | 0.090 |
|  |  |  |  | WT | 7 |  | cGMP |  | 0.049 |
| G141 |  | S |  | WT | 7 |  | cAMP |  | 0.409 |
|  |  |  |  | WT | 7 |  | cGMP |  | 4.952 |
|  |  | A |  | WT | 7 |  | cAMP |  | 0.019 |
|  |  | D |  | WT | 7 |  | cAMP |  | 0.695 |
|  |  |  |  | WT | 7 |  | cGMP |  | 9.704 |
|  |  | V |  | WT | 25 |  | None |  | 0.036 |
|  |  | Silent |  | WT | 30 |  | None |  | 0.046 |
| R142 |  | H |  | WT | 3 |  | None |  | 0.136 |
|  |  |  |  | WT | 7 |  | None |  | 0.110 |
|  |  |  |  | WT | 9 |  | None |  | 0.139 |
|  |  |  |  | WT | 7 |  | cAMP |  | 0.119 |
|  |  |  |  | WT | 7 |  | cGMP |  | 0.098 |
|  |  |  |  | A144E | 7 |  | None |  | 0.079 |
| A144 |  | Q |  | A144E | 7 |  | None |  | 0.009 |
|  |  | K |  | A144T | 7 |  | None |  | 0.030 |
|  |  |  |  | A144E | 7 |  | None |  | 1.113 |
|  |  | P |  | WT | 5 |  | None |  | 0.023 |
|  |  | T |  | WT | 1 |  | None |  | 0.138 |
|  |  |  |  | WT | 5 |  | None |  | 23.550 |
|  |  |  |  | WT | 9 |  | None |  | 0.111 |
|  |  |  |  | WT | 15 |  | None |  | 56.514 |
|  |  |  |  | WT | 20 |  | None |  | 85.331 |
|  |  |  |  | WT | 25 |  | None |  | 38.476 |
|  |  |  |  | WT | 30 |  | None |  | 83.973 |
|  |  |  |  | WT | 35 |  | None |  | 99.732 |
|  |  |  |  | WT | 7 |  | cAMP |  | 0.159 |
|  |  |  |  | WT | 7 |  | cGMP |  | 7.142 |
|  |  |  |  | A144T | 7 |  | None |  | 99.728 |
|  |  |  |  | A144E | 7 |  | None |  | 0.129 |
|  |  | E |  | WT | 7 |  | cAMP |  | 0.208 |
|  |  |  |  | WT | 7 |  | cGMP |  | 1.377 |
|  |  |  |  | A144T | 7 |  | None |  | 0.152 |
|  |  |  |  | A144E | 7 |  | None |  | 98.723 |
|  |  | G |  | WT | 7 |  | cGMP |  | 0.008 |
|  |  | V |  | WT | 7 |  | cAMP |  | 0.027 |
|  |  |  |  | WT | 7 |  | cGMP |  | 0.264 |
| L147 |  | Silent |  | WT | 35 |  | None |  | 0.123 |
| L148 |  | Silent |  | WT | 0 |  | None |  | 0.066 |
| A151 |  | T |  | WT | 7 |  | None |  | 0.113 |
|  |  |  |  | WT | 9 |  | None |  | 0.127 |
|  |  |  |  | WT | 25 |  | None |  | 0.101 |
|  |  |  |  | WT | 35 |  | None |  | 0.120 |
|  |  |  |  | A144T | 7 |  | None |  | 0.090 |
| P154 |  | Q |  | WT | 35 |  | None |  | 0.117 |
| M157 |  | V |  | WT | 5 |  | None |  | 0.059 |
|  |  | I |  | WT | 0 |  | None |  | 0.027 |
| T158 |  | P |  | WT | 0 |  | None |  | 0.055 |
|  |  |  |  | WT | 7 |  | cGMP |  | 0.064 |
|  |  | S |  | WT | 7 |  | cGMP |  | 0.096 |
| P160 |  | Silent |  | WT | 0 |  | None |  | 0.110 |
|  |  |  |  | WT | 3 |  | None |  | 0.098 |
|  |  |  |  | WT | 25 |  | None |  | 0.090 |
|  |  |  |  | A144T | 7 |  | None |  | 0.096 |
| D161 |  | G |  | WT | 9 |  | None |  | 0.142 |
|  |  | E |  | WT | 35 |  | None |  | 0.039 |
|  |  | Silent |  | WT | 35 |  | None |  | 0.156 |
| T168 |  | S |  | WT | 35 |  | None |  | 0.078 |
| Q170 |  | E |  | A144T | 7 |  | None |  | 0.032 |
|  |  | K |  | WT | 30 |  | None |  | 82.890 |
|  |  |  |  | WT | 35 |  | None |  | 1.404 |
|  |  |  |  | WT | 7 |  | cAMP |  | 0.421 |
|  |  |  |  | A144T | 7 |  | None |  | 0.226 |
|  |  |  |  | A144E | 7 |  | None |  | 3.669 |
| Q174 |  | P |  | WT | 9 |  | None |  | 0.070 |
| R180 |  | G |  | WT | 30 |  | None |  | 0.055 |
| L187 |  | R |  | WT | 35 |  | None |  | 0.039 |
| M189 |  | R |  | WT | 7 |  | cGMP |  | 0.095 |
|  |  |  |  | A144T | 7 |  | None |  | 0.097 |
|  |  |  |  | A144E | 7 |  | None |  | 1.197 |
|  |  | K |  | WT | 30 |  | None |  | 7.072 |
|  |  |  |  | WT | 7 |  | cGMP |  | 0.063 |
|  |  |  |  | A144E | 7 |  | None |  | 1.160 |
| D192 |  | A |  | A144T | 7 |  | None |  | 0.097 |
| Q193 |  | K |  | WT | 15 |  | None |  | 24.056 |
| L195 |  | R |  | WT | 7 |  | cAMP |  | 0.065 |
|  |  |  |  | WT | 7 |  | cGMP |  | 5.566 |
|  |  | Q |  | WT | 7 |  | cAMP |  | 0.324 |
| A198 |  | T |  | WT | 7 |  | None |  | 0.166 |
|  |  |  |  | WT | 15 |  | None |  | 0.179 |
| H199 |  | D |  | WT | 20 |  | None |  | 0.082 |
| G200 |  | S |  | A144E | 7 |  | None |  | 0.183 |
| Y206 |  | Silent |  | A144T | 7 |  | None |  | 0.193 |

**References**

Aiba, H., Nakamura, T., Mitani, H., Mori, H., 1985. Mutations that alter the allosteric nature of cAMP receptor protein of Escherichia coli. EMBO J. 4, 3329–3332. doi:10.1002/j.1460-2075.1985.tb04084.x

Belduz, A.O., Lee, E.J., Harman, J.G., 1993. Mutagenesis of the cyclic AMP receptor protein of Escherichia coli : targeting positions 72 and 82 of the cyclic nucleotide binding pocket. Nucleic Acids Res. 21, 2894-1827–1835. doi:10.1093/nar/22.15.2894

Bell, A., Gaston, K., Williams, R., Chapman, K., Kolb, A., Buc, H., Minchin, S., Williams, J., Busby, S., 1990. Mutations that alter the ability of the Escherichia coli cyclic AMP receptor protein to activate transcription. Nucleic Acids Res. 18, 7243–50. doi:10.1093/nar/18.24.7243

Breul, A., Aßmann, H., Golz, R., von Wilcken-Bergmann, B., Müller-Hill, B., 1993. Mutants with substitutions for Glu171 in the catabolite activator protein (CAP) of Escherichia coli activate transcription from the lac promoter. MGG Mol. Gen. Genet. 238, 155–160. doi:10.1007/BF00279542

Cheng, X., Ching Lee, J., 1998. Interactive and dominant effects of residues 128 and 141 on cyclic nucleotide and DNA bindings in Escherichia coli cAMP receptor protein. J. Biol. Chem. 273, 705–712. doi:10.1074/jbc.273.2.705

Cheng, X., Kovac, L., Ching Lee, J., 1995. Probing the Mechanism of CRP Activation by Site-Directed Mutagenesis: The Role of Serine 128 in the Allosteric Pathway of cAMP Receptor Protein Activation. Biochemistry 34, 10816–10826. doi:10.1021/bi00034a014

Cheng, X., Lee, J.C., 1994. Absolute requirement of cyclic nucleotide in the activation of the G141Q mutant cAMP receptor protein from Escherichia coli. J. Biol. Chem. 269, 30781–30784.

Conrad, T.M., Joyce, A.R., Applebee, M.K., Barrett, C.L., Xie, B., Gao, Y., Palsson, B.T., 2009. Whole-genome resequencing of Escherichia coli K-12 MG1655 undergoing short-term laboratory evolution in lactate minimal media reveals flexible selection of adaptive mutations. Genome Biol. 10, 1–12. doi:10.1186/gb-2009-10-10-r118

Dai, J., Lin, S.-H., Kemmis, C., Chin, A.J., Lee, J.C., 2004. Interplay between Site-Specific Mutations and Cyclic Nucleotides in Modulating DNA Recognition by Escherichia coli Cyclic AMP Receptor Protein † , ‡. Biochemistry 43, 8901–8910. doi:10.1021/bi0499359

Eschenlauer, A.C., Reznikoff, W.S., 1991. Escherichia coli catabolite gene activator protein mutants defective in positive control of lac operon transcription. J. Bacteriol. 173, 5024–5029. doi:10.1128/jb.173.16.5024-5029.1991

Garges, S., Adhya, S., 1988. Cyclic AMP-induced conformational change of cyclic AMP receptor protein (CRP): intragenic suppressors of cyclic AMP-independent CRP mutations. J. Bacteriol. 170, 1417–1422. doi:10.1128/jb.170.4.1417-1422.1988

Garges, S., Adhya, S., 1985. Sites of allosteric shift in the structure of the cyclic AMP receptor protein. Cell 41, 745–751. doi:10.1016/S0092-8674(85)80055-6

Gorshkova, I., Moore, J.L., McKenney, K.H., Schwarz, F.P., 1995. Thermodynamics of cyclic nucleotide binding to the cAMP receptor protein and its T127L mutant. J. Biol. Chem. 270, 21679–21683. doi:10.1074/jbc.270.37.21679

Gronenborn, A.M., Sandulache, R., Gärtner, S., Clore, G.M., 1988. Mutations in the cyclic AMP binding site of the cyclic AMP receptor protein of Escherichia coli. Biochem. J. 253, 801–807. doi:10.1042/bj2530801

Gunasekara, S.M., Hicks, M.N., Park, J., Brooks, C.L., Serate, J., Saunders, C. V., Grover, S.K., Goto, J.J., Lee, J.-W., Youn, H., 2015. Directed Evolution of the Escherichia coli cAMP Receptor Protein at the cAMP Pocket. J. Biol. Chem. 290, 26587–26596. doi:10.1074/jbc.m115.678474

Harman, J.G., McKenney, K., Peterkofsky, A., 1986. Structure-function analysis of three cAMP-independent forms of the cAMP receptor protein. J. Biol. Chem. 261, 16332–16339. doi:3023348

Harman, J.G., Peterkofsky, A., McKenney, K., 1988. Arginine substituted for leucine at position 195 produces a cyclic AMP-independent form of the Escherichia coli cyclic AMP receptor protein. J. Biol. Chem. 263, 8072–8077.

Irwin, N., Ptashne, M., 1987. Mutants of the catabolite activator protein of Escherichia coli that are specifically deficient in the gene-activation function. Proc. Natl. Acad. Sci. 84, 8315–8319. doi:10.1073/pnas.84.23.8315

Kim, J., Adhya, S., Garges, S., 1992. Allosteric changes in the cAMP receptor protein of Escherichia coli: hinge reorientation. Proc. Natl. Acad. Sci. 89, 9700–9704. doi:10.1073/pnas.89.20.9700

Latif, H., Federowicz, S., Ebrahim, A., Tarasova, J., Szubin, R., Utrilla, J., Zengler, K., Palsson, B.O., 2018. ChIP-exo interrogation of Crp, DNA, and RNAP holoenzyme interactions. PLoS One 13, e0197272. doi:10.1371/journal.pone.0197272

Lee, E.J., Glasgow, J., Leu, S.-F., Belduz, A.O., Harman, J.G., 1994. Mutagenesis of the cyclic AMP receptor protein of Escherichia coli : targeting positions 83, 127 and 128 the cyclic nucleotide binding pocket. Nucleic Acids Res. 22, 2894–2901. doi:10.1093/nar/22.15.2894

Lin, S.H., Kovac, L., Chin, A.J., Chin, C.C.Q., Lee, J.C., 2002. Ability of E. coli Cyclic AMP receptor protein to differentiate cyclic nucelotides: Effects of single site mutations. Biochemistry 41, 2946–2955. doi:10.1021/bi0119215

Macconkey, A., 1905. Lactose-fermenting bacteria in faeces. J. Hyg. (Lond).

Melton, T., Snow, L.L., Freitag, C.S., Dobrogosz, W.J., 1981. Isolation and Characterization of cAMP Suppressor Mutants of Escherichia coli K12. Mol. Gen. Genet. 182, 480–489.

Moore, J., Kantorow, M., Vanderzwaag, D., McKenney, K., 1992. Escherichia coli Cyclic AMP Receptor Protein Mutants Provide Evidence for Ligand Contacts Important in Activation. J. Bacteriol. 174, 8030–8035. doi:10.1128/jb.174.24.8030-8035.1992

Moore, J.L., Gorshkova, I.I., McKenney, K.H., Schwarz, F.P., Brown, J.W., 1996. Effect of cAMP Binding Site Mutations on the Interaction of cAMP Receptor Protein with Cyclic Nucleoside Monophosphate Ligands and DNA. J. Biol. Chem. 271, 21273–21278. doi:10.1074/jbc.271.35.21273

Niu, W., Kim, Y., Tau, G., Heyduk, T., Ebright, R.H., 1996. Transcription activation at class II CAP-dependent promoters: Two interactions between CAP and RNA polymerase. Cell 87, 1123–1134. doi:10.1016/S0092-8674(00)81806-1

Puskas, R., Fredd, N., Gazdar, C., Peterkofsky, A., 1983. Methylglyoxal-Mediated Growth Inhibition in an Escherichia coli cAMP Receptor Protein Mutant. Arch. Biochem. Biophys. 223, 503–513.

Rhodius, V.A., Busby, S.J.W., 2000. Interactions between Activating Region 3 of the Escherichia coli cyclic AMP receptor protein and region 4 of the RNA polymerase σ70subunit: Application of suppression genetics. J. Mol. Biol. 311–324. doi:10.1006/jmbi.2000.3737

Rhodius, V.A., West, D.M., Webster, C.L., Busby, S.J.W., Savery, N.J., 1997. Transcription activation at class II CRP-dependent promoters: The role of different activating regions. Nucleic Acids Res. 25, 326–332. doi:10.1093/nar/25.2.326

Ryu, S., Kim, J., Adhya, S., Garges, S., 1993. Pivotal role of amino acid at position 138 in the allosteric hinge reorientation of cAMP receptor protein. Proc. Natl. Acad. Sci. 90, 75–79. doi:10.1073/pnas.90.1.75

Sanders, R., McGeoch, D., 1973. A Mutant Transcription Factor That is Activated By 3’:5’-Cyclic Guanosine Monophosphat. Proc. Natl. Acad. Sci. 70, 1017–1021.

Sekowska, A., Wendel, S., Fischer, E.C., Nørholm, M.H.H., Danchin, A., 2016. Generation of mutation hotspots in ageing bacterial colonies. Sci. Rep. 6, 2. doi:10.1038/s41598-016-0005-4

Sievert, C., Nieves, L.M., Panyon, L.A., Loeffler, T., Morris, C., Cartwright, R.A., Wang, X., 2017. Experimental evolution reveals an effective avenue to release catabolite repression via mutations in XylR. Proc. Natl. Acad. Sci. 114, 7349–7354. doi:10.1073/pnas.1700345114

Søgaard-Andersen, L., Mironov, A.S., Pedersen, H., Sukhodelets, V. V., Valentin-Hansen, P., 1991. Single amino acid substitutions in the cAMP receptor protein specifically abolish regulation by the CytR repressor in Escherichia coli. Proc. Natl. Acad. Sci. 88, 4921–4925. doi:10.1073/pnas.88.11.4921

Tagami, H., Inada, T., Kunimura, T., Aiba, H., 1995. Glucose lowers CRP*levels resulting in repression of the lac operon in cells lacking cAMP. Mol. Microbiol. 17, 251–258. doi:10.1111/j.1365-2958.1995.mmi_17020251.x

Tzeng, S.R., Kalodimos, C.G., 2009. Dynamic activation of an allosteric regulatory protein. Nature 462, 368–372. doi:10.1038/nature08560

Wang, S., Shi, Y., Gorshkova, I., Schwarz, F.P., 2000. RNA polymerase-cNMP-ligated cAMP receptor protein (CRP) mutant interactions in the enhancement of transcription by CRP mutants. J. Biol. Chem. 275, 33457–33463. doi:10.1074/jbc.M004877200

West, D., Williams, R., Rhodius, V., Bell, A., Sharma, N., Zou, C., Fujita, N., Ishihama, A., Busby, S., 1993. Interactions between the Escherichia coli cyclic AMP receptor protein and RNA polymerase at Class II promoters. Mol. Microbiol. 10, 789–797. doi:10.1111/j.1365-2958.1993.tb00949.x

Williams, R.M., Rhodius, V.A., Bell, A.I., Kolb, A., Busby, S.J.W., 1996. Orientation of functional activating regions in the Escherichia coli CRP protein during transcription activation at class II promoters. Nucleic Acids Res. 24, 1112–1118. doi:10.1093/nar/24.6.1112

Youn, H., Kerby, R.L., Conrad, M., Roberts, G.P., 2006. Study of highly constitutively active mutants suggests how cAMP activates cAMP receptor protein. J. Biol. Chem. 281, 1119–1127. doi:10.1074/jbc.M509421200

Zhang, X., Zhou, Y., Ebright, W., Ebrights, H., 1992. Catabolite Gene Activator Protein (CAP) Is Not an “ Acidic Activating Region ” Transcription Activator Protein. J. Biol. Chem. 267, 8136–8139.

Zhang, X.P., Ebright, R.H., 1990. Identification of a contact between arginine-180 of the catabolite gene activator protein (CAP) and base pair 5 of the DNA site in the CAP-DNA complex. Proc. Natl. Acad. Sci. 87, 4717–4721. doi:10.1073/pnas.87.12.4717

Zhou, Y., Zhang, X., Ebright, R.H., 1993. Identification of the activating region of catabolite gene activator protein (CAP): isolation and characterization of mutants of CAP specifically defective in transcription activation. Proc. Natl. Acad. Sci. U. S. A. 90, 6081–5.
